# Characterization of immune response to neurofilament light in experimental autoimmune encephalomyelitis

**DOI:** 10.1186/1742-2094-10-118

**Published:** 2013-09-22

**Authors:** Fabiola Puentes, Baukje J van der Star, Marion Victor, Markus Kipp, Cordian Beyer, Regina Peferoen-Baert, Kimberley Ummenthum, Gareth Pryce, Wouter Gerritsen, Ruth Huizinga, Arie Reijerkerk, Paul van der Valk, David Baker, Sandra Amor

**Affiliations:** 1 Neuroimmunology Unit, Blizard Institute, Bart’s and the London School of Medicine and Dentistry, Queen Mary University of London, London E1 2AT, UK; 2 Pathology Department, VU University Medical Center, Amsterdam, The Netherlands, De Boelelaan 1117, 1081 HV, Amsterdam, The Netherlands; 3 Institute of Neuroanatomy, Faculty of Medicine, RWTH Aachen University, Aachen 52062, Germany; 4 Department of Immunology, Erasmus MC, University Medical Center, Rotterdam 3062 PA, The Netherlands; 5 Molecular Cell Biology and Immunology Department, VU Medical Center, Amsterdam, The Netherlands, De Boelelaan 1117, 1081 HV, Amsterdam, The Netherlands

**Keywords:** Neurofilament light, Axonal damage, Neurodegeneration, Experimental autoimmune encephalomyelitis, Multiple sclerosis

## Abstract

**Background:**

Autoimmunity to neuronal proteins occurs in several neurological syndromes, where cellular and humoral responses are directed to surface as well as intracellular antigens. Similar to myelin autoimmunity, pathogenic immune response to neuroaxonal components such as neurofilaments may contribute to neurodegeneration in multiple sclerosis.

**Methods:**

We studied the immune response to the axonal protein neurofilament light (NF-L) in the experimental autoimmune encephalomyelitis animal model of multiple sclerosis. To examine the association between T cells and axonal damage, pathology studies were performed on NF-L immunized mice. The interaction of T cells and axons was analyzed by confocal microscopy of central nervous system tissues and T-cell and antibody responses to immunodominant epitopes identified in ABH (H2-A^g7^) and SJL/J (H2-A^s^) mice. These epitopes, algorithm-predicted peptides and encephalitogenic motifs within NF-L were screened for encephalitogenicity.

**Results:**

Confocal microscopy revealed both CD4^+^ and CD8^+^ T cells alongside damaged axons in the lesions of NF-L immunized mice. CD4^+^ T cells dominated the areas of axonal injury in the dorsal column of spastic mice in which the expression of granzyme B and perforin was detected. Identified NF-L epitopes induced mild neurological signs similar to the observed with the NF-L protein, yet distinct from those characteristic of neurological disease induced with myelin oligodendrocyte glycoprotein.

**Conclusions:**

Our data suggest that CD4^+^ T cells are associated with spasticity, axonal damage and neurodegeneration in NF-L immunized mice. In addition, defined T-cell epitopes in the NF-L protein might be involved in the pathogenesis of the disease.

## Introduction

Multiple sclerosis (MS) is a chronic demyelinating and neurodegenerative disease of the central nervous system (CNS) widely considered due to aggressive, autoreactive T cells and antibodies to myelin [1-3]. However, accumulating evidence shows that immune responses to neuronal and axonal proteins are also present in a wide range of neurodegenerative disorders including MS [4-6]. That these responses may contribute to axonal and neuronal damage, pathological hallmarks of MS, is supported by observations that immunization with neuronal antigens and transfer of antibodies directed to neuronal and axonal proteins induce neuronal damage in animals [7-10].

The lack of expression of molecules of the major histocompatibility complex (MHC) class II on neurons indicates that neurons cannot activate CD4^+^ cells in an antigen-specific manner. However, neurons constitutively express or readily upregulate expression of MHC class I during inflammation, indicating that neurons may become targets for CD8^+^ T cells [11]. Conceptually, both CD4^+^ and CD8^+^ T cells could mediate attack on axons and neurons, either by direct contact via antigen-independent interactions or as a result of collateral damage [12]. Activated T cells in the CNS are reported to produce cytotoxic molecules as well as glutamate, nitric oxide and reactive oxygen species that could contribute to the damage and progressive neurodegeneration observed in MS and other neurodegenerative diseases in which inflammation has been described [13-15]. In addition, activation of B cells might lead to the production of specific antibodies to neuronal antigens that could also contribute to the damage and progressive neurodegeneration [16].

To examine the mechanisms of autoimmunity to neurons we have developed a model of autoimmune induced axonal and neuronal damage following immunization of mice with the neuronal cytoskeletal protein neurofilament light (NF-L) [7]. Whether T-cell responses to neuroaxonal components are pathogenic in MS is as yet unknown; although we have recently shown that NF-L is phagocytosed by MHC class II^+^ microglia/macrophages in MS brain lesions [17], indicating a potential source by which autoreactive T cells could become reactivated in MS. In mice, we have shown that autoimmunity to NF-L causes spasticity and neurodegeneration and that axonal damage is a direct consequence of such responses [7,8]. Infiltration of CD3^+^ T cells and B220^+^ cells mainly localized in the dorsal column of the spinal cord of NF-L immunized mice was also observed [8]. Likewise, immunoglobulin deposits were observed into the axons in mice immunized with NF-L protein [7].

In the present study, we characterized the T-cell infiltrates in the CNS of spastic mice. Both CD4^+^ and CD8^+^ T cells were found in close association to axons, although CD4^+^ T cells dominated the infiltrates in lesions. In addition, increased perforin expression and cells positive for granzyme B could be observed in the spinal cord of mice immunized with NF-L. Furthermore, NF-L peptides were screened for T-cell and B-cell responses in ABH (H2-A^g7^) and SJL/J (H2-A^s^) mice, and the pathogenic potential of these peptides and predicted binding motifs to H2-A^g7^ present in the NF-L sequence were investigated.

In summary, our study reveals that T cells associated with the expression of cytotoxic molecules are present in lesions in the dorsal columns of spastic mice immunized with NF-L and we show, for the first time, that active immunization with defined NF-L peptides induced neurological disease in ABH mice.

## Materials and methods

### Mice

Male and female 10-week-old Biozzi ABH (H-2^dq1^) and SJL/J (H-2^s^) mice were obtained from Harlan (Bicester, UK) and Charles River laboratories (Kent, UK) or were bred at QMUL (London, UK). All procedures were performed in accordance with the UK Animals (Scientific Procedures) Act (1986) and approved by the local ethics committee. All procedures were performed following Institutional ethical review in accordance to the United Kingdom Animals (Scientific Procedures) Act (1986) and European Union Directive 2010/63/EU. Animals were housed and monitored consistent with the principles of the ARRIVE guidelines as described previously [18].

### Antigens

Spinal cord homogenate (SCH) prepared from 60 ABH mice was lyophilized and reconstituted in PBS as described previously [18]. rmNF-L was prepared as described previously [17]. Overlapping 16-amino-acid peptides (Table 1) based on the mouse protein sequence (NCBI protein ID: NP035040) and myelin oligodendrocyte glycoprotein (MOG^35–55^) peptide (MEVGWYRSPFSRVVHLYRNGK) were synthesized as peptide amides (CONH_2_) (Cambridge Research Biochemicals Ltd, Billingham, UK).

**Table 1:** **Sequences of mouse neurofilament-light peptides**^**a**^

**Sequence**	**Amino acid**	**Sequence**	**Amino acid**
1 to 16	SSFGYDPYFSTSYKRR	273 to 288	MQNAEEWFKSRFTVLT
9 to 24	FSTSYKRRYVETPRVH	281 to 296	KSRFTVLTESAAKNTD
17 to 32	YVETPRVHISSVRSGY	289 to 304	ESAAKNTDAVRAAKDE
25 to 40	ISSVRSGYSTARSAYS	297 to 312	AVRAAKDEVSESRRLL
33 to 48	STARSAYSSYSAPVSS	305 to 320	VSESRRLLKAKTLEIE
41 to 56	SYSAPVSSSLSVRRSY	313 to 328	KAKTLEIEACRGMNEA
49 to 64	SLSVRRSYSSSSGSLM	321 to 336	ACRGMNEALEKQLQEL
57 to 72	SSSSGSLMPSLENLDL	329 to 344	LEKQLQELEDKQNADI
65 to 80	PSLENLDLSQVAAISN	337 to 352	EDKQNADISAMQDTIN
73 to 88	SQVAAISNDLKSIRTQ	345 to 360	SAMQDTINKLENELRS
81 to 96	DLKSIRTQEKAQLQDL	353 to 368	KLENELRSTKSEMARY
89 to 104	EKAQLQDLNDRFASFI	361 to 376	TKSEMARYLKEYQDLL
97 to 112	NDRFASFIERVHELEQ	369 to 384	LKEYQDLLNVKMALDI
105 to 120	ERVHELEQQNKVLEAE	377 to 392	NVKMALDIEIAAYRKL
113 to 128	QNKVLEAELLVLRQKH	385 to 400	EIAAYRKLLEGEETRL
121 to 136	LLVLRQKHSEPSRFRA	393 to 408	LEGEETRLSFTSVGSI
129 to 144	SEPSRFRALYEQEIRD	401 to 416	SFTSVGSITSGYSQSS
137 to 152	LYEQEIRDLRLAAEDA	409 to 424	TSGYSQSSQVFGRSAY
145 to 160	LRLAAEDATNEKQALQ	417 to 432	QVFGRSAYSGLQSSSY
153 to 168	TNEKQALQGEREGLEE	425 to 440	SGLQSSSYLMSARSFP
161 to 176	GEREGLEETLRNLQAR	433 to 448	LMSARSFPAYYTSHVQ
169 to 184	TLRNLQARYEEEVLSR	441 to 456	AYYTSHVQEEQTEVEE
177 to 192	YEEEVLSREDAEGRLM	449 to 464	EEQTEVEETIEATKAE
185 to 200	EDAEGRLMEARKGADE	457 to 472	TIEATKAEEAKDEPPS
193 to 208	EARKGADEAALARAEL	465 to 480	EAKDEPPSEGEAEEEE
201 to 216	AALARAELEKRIDSLM	473 to 488	EGEAEEEEKEKEEGEE
209 to 224	EKRIDSLMDEIAFLKK	481 to 496	KEKEEGEEEEGAEEEE
217 to 232	DEIAFLKKVHEEEIAE	489 to 504	EEGAEEEEAKDESEDT
225 to 240	VHEEEIAELQAQIQYA	497 to 512	AKDESEDTKEEEEGGE
233 to 248	LQAQIQYAQISVEMDV	505 to 520	KEEEEGGEGEEEDTKE
241 to 256	QISVEMDVSSKPDLSA	513 to 528	GEEEDTKESEEEEKKE
249 to 264	SSKPDLSAALKDIRAQ	521 to 536	SEEEEKKEESAGEEQV
257 to 272	ALKDIRAQYEKLAAKN	529 to 544	ESAGEEQVAKKKD
265 to 280	YEKLAAKNMQNAEEWF		

^a^Mouse neurofilament-light sequence (NCBI reference sequence: NP035040).

### Induction of experimental autoimmune encephalomyelitis

Mice were injected subcutaneously with 1 mg SCH, 200 μg rmNF-L, 200 μg MOG^35–55^ or rmNF-L and MOG^35–55^ (1:1), or pools of 30 μg each peptide emulsified with incomplete Freund’s adjuvant (Difco Laboratories, (Detroit, Michigan, USA) supplemented with 48 μg *Mycobacterium tuberculosis* and 6 μg *Mycobacterium butyricum* (Difco Laboratories) on day 0 and day 7 as described previously [19]. Control mice were immunized with complete Freund’s adjuvant (CFA) only. All mice were injected with 200 ng *Bordetella pertussis* toxin (Sigma St. Louis, Missouri, USA) intraperitoneally, immediately after immunization and 24 hours later.

To identify encephalitogenic epitopes, four to six mice were immunized with rmNF-L, individual or pooled peptides. To optimize identification, sequences containing motifs that bind to or interact with H2-A^g7^ were selected as described previously [19]. The Rankpep server was additionally used to predict binding to H2-A^g7^[20].

Mice were monitored daily and scored according to a neurological scale: 0, normal; 0.5, partial loss of tail tone; 1, paralysis or spasticity of the tail; 2, impaired righting reflex; 3, paralysis or spastic paresis of one limb; 4, paralysis or spastic paresis of two limbs; and 5, moribund [7,18]. Mice were sacrificed by carbon dioxide inhalation and brains and spinal cords snap-frozen in liquid nitrogen or processed for pathology [7].

### Immunohistochemistry

Sections (3 μm) from snap-frozen spinal cord tissues were fixed with acetone and incubated overnight at 4°C with mAb directed to CD4 (YTS 191.1.2), CD8 (YTS 169AG; ImmunoTools, Friesoythe, Germany), MHC-I antigens (HM1091; Hycult Biotech, Plymouth Meeting, PA, USA) or biotinylated MHC-II (OX 6, a kind gift of Jack van Horssen, VU University Medical Center) diluted in antibody diluent (Immunologic; Duiven, The Netherlands). After washing, endogenous peroxidase was blocked with 0.3% H_2_O_2_ in PBS. Sections stained for CD4, CD8 and MHC-I were incubated with biotinylated rabbit anti-rat Ig (Dako, Glostrup, Denmark) for 1 hour, followed by peroxidase-coupled avidin–biotin complex (ABC kit; Vector Laboratories, Burlingame, CA, USA). Sections stained with biotinylated MHC-II were incubated with streptavidin–horseradish peroxidase complex (Dako) for 1 hour. All secondary antibodies were visualized with 3,3′-diaminobenzidine. Antibodies were prescreened on brain, liver, lung, spleen and tonsil tissues and isotype control mAb served as negative control. The percentage of CD4^+^ and CD8^+^ T cells were counted at 25× objective at three levels of the spinal cord.

For immunofluorescence, sections were incubated with blocking solution (CleanVision IHC/ICC; Immunologic) containing 10% normal goat serum for 2 hours, washed in PBS and incubated with mAb to NF-L (10H9), SMI-32 (Covance, Princeton, NJ, USA) or NeuN (Merck Millipore; Darmstadt, Germany) and CD3 (CD3-12; Serotec, Oxford, UK), CD4 (YTS 191.1.2) or CD8 (YTS 169AG; ImmunoTools) overnight at 4°C. After washing in PBS, sections were incubated with goat anti-mouse IgG1 Alexa 594 or goat anti-rat IgG Alexa 488 (Invitrogen; Paisley, UK) for 60 minutes at room temperature. Sections were viewed using confocal laser scanning microscopy (Leica DMI6000; Rijswijk, The Netherlands). Image processing was performed using NIH Image J software [21].

Granzyme B staining was performed on paraffin-embedded sections (4 μm). In brief, sections were deparaffinized and rinsed in H_2_O. Subsequently, endogenous peroxidase was blocked as described above. After rinsing in PBS, antigen retrieval in Tris–ethylenediamine tetraacetic acid buffer (pH 9.0) was performed in a microwave followed by incubation with 10% normal goat serum. Sections were incubated overnight at 4°C with polyclonal rabbit anti-granzyme B (ab4059; Abcam; Cambridge, UK) in 1% BSA. Subsequently, sections were washed in PBS and incubated with secondary antibody Envision anti Rabbit labeled with horseradish peroxidase (K4002; Dako) for 30 minutes and visualized with 3,3′-diaminobenzidine.

### Reverse transcriptase polymerase chain reaction

Spinal cords from control and NF-L immunized ABH mice were dissolved in lysis buffer (NucleoSpin RNA/Protein kit; Machery-Nagel GmbH, Düren, Germany) and homogenized with 1.4 mm ceramic beads (Precellys 24; Peqlab Biotechnologie GmbH, Erlangen, Germany) at 5000 rpm for 15 seconds. Subsequently, RNA was isolated using NuceloSpin (Macherey-Nagel) according to the manufacturer’s recommendations. Purity was confirmed using 260:280 OD ratios (Nano-Drop 1000; Peqlab Biotechnologie GmbH). RT reactions were performed with the MMLV RT-kit and random hexanucleotide primers (Invitrogen) and gene expression was measured using Taq-Polymerase (Biomol GmbH, Hamburg, Germany).

Primers for perforin amplification (sense, 5′-CTGCCACTCGGTCAGAATG-3′; antisense, 5′-CGGAGGGTAGTCACATCCAT-3′) were used at annealing temperature of 59°C, amplifying an 88-base-pair fragment. Expression levels of the reference gene hypoxanthine guanine phosphoribosyl transferase were used as control. Primers for hypoxanthine guanine phosphoribosyl transferase amplification (sense, 5′-GCTGGTGAAAAGGACCTCT-3′; antisense: 5′-CACAGGACTAGAACACCTGC-3′) were used at an annealing temperature of 60°C, amplifying a 248-base-pair fragment, and primers for 18sRNA amplification (sense, 5′-CGGCTACCACATCCAAGGAA-3′; antisense, 5′-GCTGGAATTACCGCGGCT-3′) were used at an annealing temperature of 60°C, amplifying a 187-base-pair fragment. Gene expression was performed using RT-PCR technology (Bio-Rad; Munich, Germany) with SYBR green (SensiMix™; Bioline; Luckenwalde, Germany), as published previously [22].

### T-cell proliferation assays

ABH and SJL/J mice were immunized with rmNF-L in CFA or CFA only (*n* = 4 per group). Spleen cells were collected 10 days after priming and single-cell suspensions (3 × 10^5^/ml) cultured in RPMI medium (Gibco, Invitrogen; Paisley, UK) with 5% FCS (Gibco, Invitrogen), 2 mM l-glutamine, 100 U/ml penicillin, 100 μg/ml streptomycin, 5 mM Hepes, and 5 × 10^–5^ M 2-mercaptoethanol (Gibco, Invitrogen). Cells were incubated with NF-L peptides or rmNF-L for 72 hours. Proliferation was determined by incorporation of [^3^H]-thymidine (GE Healthcare, Uppsala, Sweden). Stimulation indices (SIs) were calculated as the proliferative response in the presence of antigen divided by the response in the absence of antigen. SI in the CFA control group ranged from 0.6 to 1.1. Positive stimulation was defined as SI >1.5.

### Enzyme-linked immunosorbent assay

To identify B-cell epitopes in NF-L, Biozzi ABH and SJL/J mice (*n* = 4 per group) were immunized with rmNF-L protein in complete adjuvant and serum was collected on day 15 post immunization. The mouse immune sera were tested for their reactivity to NF-L overlapping peptides. Nunclon plates (Nunc, Roskilde, Denmark) were coated overnight at 4°C with 10 μg/ml mouse NF-L peptides or rmNF-L protein, in carbonate buffer. Plates were washed twice in PBS and blocked for 1 hour at 37°C with 2% BSA/PBS. After blocking, ABH or SJL/J immune sera, diluted 1:100 in 1% (BSA/PBS), were added and incubated for 1 hour at room temperature. After washing in PBS–Tween 0.1%, the plates were incubated for 1 hour at room temperature with horseradish peroxidase-conjugated rabbit anti-mouse Ig (Dako). The reaction product was developed with TMB substrate (Thermo Fisher Scientific; Loughborough, UK) and stopped by the addition of 2 M hydrochloric acid. The absorbance was measured at 450 nm, using a Synergy HT microplate reader (Bio-Tek instruments; Winooski, Vermont, USA). Background values were obtained by the reactivity of immune serum on peptide uncoated wells. An absorbance above the mean plus three standard deviations of the background reactivity was taken as positive.

### Statistical analysis

For comparison of clinical experimental autoimmune encephalomyelitis (EAE) scores, significance between the groups was determined by nonparametric Mann–Whitney rank-sum test (SigmaStat; Systat Software Inc., San Jose, CA, USA). Data represent the mean ± standard error of the mean (**P <0.01, *P <0.05).

## Results

### CD4^+^ and CD8^+^ T cells are associated with neuronal/axonal damage in spastic mice

**Figure 1:**
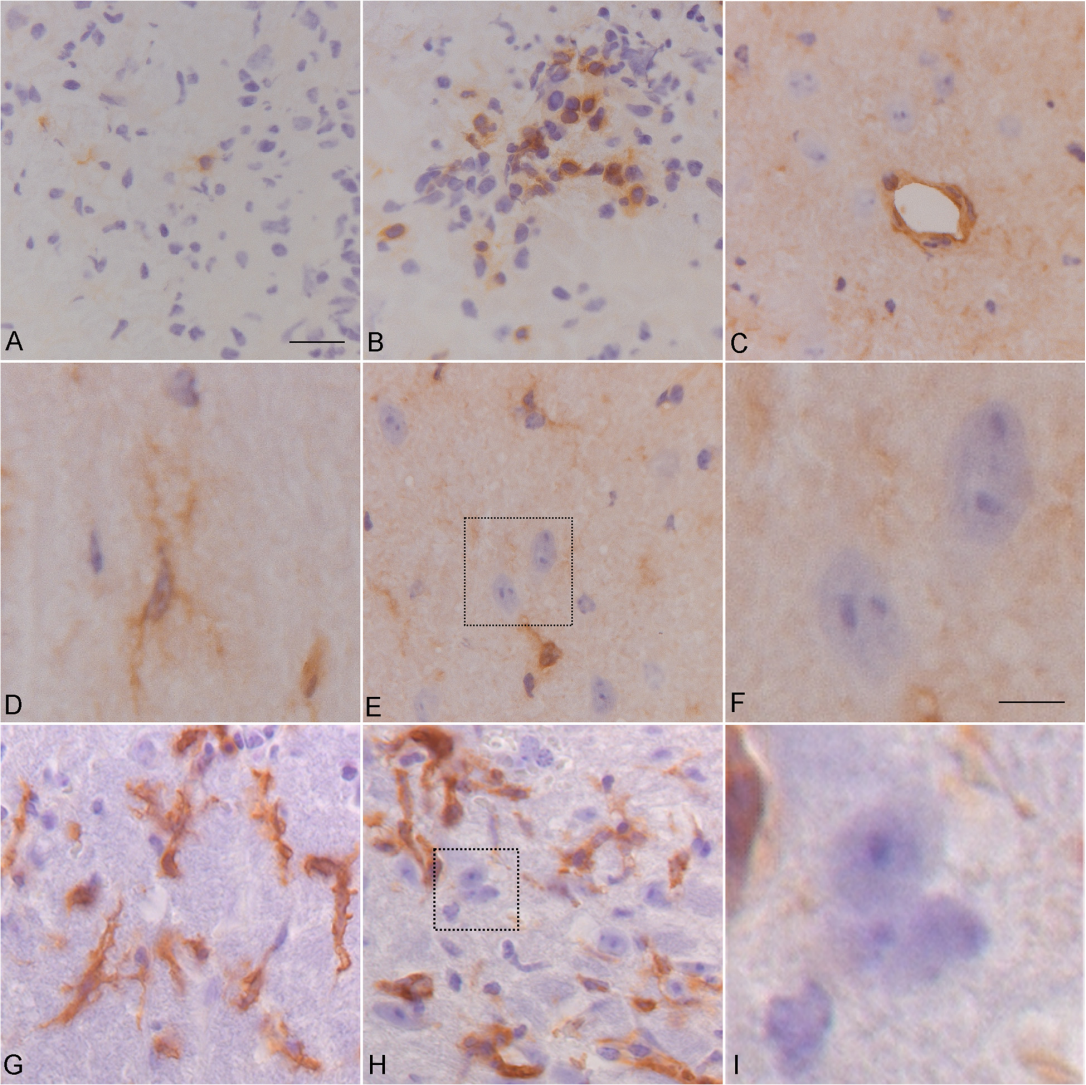
**T cells in lesions of spastic mice.** Immunohistochemistry showing **(A)** CD8^+^ and **(B)** CD4^+^ T cells in the dorsal column of a spastic mouse. **(C)** to **(F)** Major histocompatibility complex (MHC) class I antigen staining expressed on **(C)** endothelial cells, and **(D)** microglia **(E**, **F)**, but not on neurons. **(G)** to **(I)** MHC class II staining on ramified cells resembling microglia but not neurons **(H**, **I)**. **A** to **E**, **G** to **H**, bar = 10 μm; **F** and **I**, bar = 5 μm.

To examine the presence of T cells in the CNS during disease, CD4^+^ or CD8^+^ T cells within the lesions of NF-L immunized mice at the peak of disease were examined. Using immunohistochemistry, the ratio of CD4^+^ and CD8^+^ T cells in the lesions was determined (Figure 1A, B, respectively). We observed that significantly more CD4^+^ T cells were present within the lesions in a ratio of 9:1 CD4^+^:CD8^+^ T cells (data not shown). To support a role for CD8^+^ T cells we examined the expression of MHC class I in the spinal cord of diseased mice. In contrast to expression of MHC class I antigens on inflammatory cells in the lesion as well as perivascular cells (Figure 1C) and microglia in normal-appearing white matter (Figure 1D), there was no evidence of MHC-class I antigen expression on neurons or axons in the lesions (Figure 1E, F) or in normal-appearing tissues (data not shown). Likewise, while MHC class II was expressed on inflammatory cells in the blood vessels and in the parenchyma, being present on ramified cells resembling microglia (Figure 1G), no expression on neurons and axons was observed (Figure 1H, I), as has been shown before in normal or pathological tissues during EAE [23].

**Figure 2:**
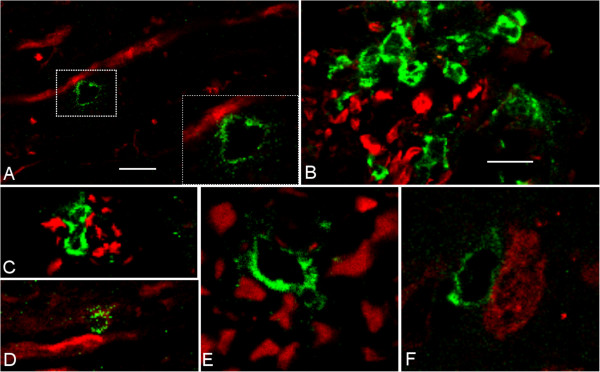
**T cells associated with axonal damage in spastic mice.** Confocal images of **(A)** CD3^+^ T cell (green) in contact with SMI32^+^ axon (red). **(B, C)** CD4^+^ T cells in area of axonal damage and **(D)** next to swollen NF-L^+^ axons. **(E)** CD8^+^ T cells between NF-L^+^ axons and **(F)** Neu-N^+^ neurons in the spinal cord. **A**, **E**, **F**, bar = 5 μm; **B**, **C**, **D**, bar = 25 μm.

To examine the interaction of T cells and axons we used confocal microscopy of CNS tissues from spastic mice. We observed a close association of CD3^+^ T cells aligning with possible damaged axons as indicated by SMI-32 staining (Figure 2A), in which there was close association between the T cell and the axon (Figure 2A, insert). In cross-sections of the lesion, CD4^+^ T cells were observed within areas of axonal damage (Figure 2B) and frequently observed between axons with increased immunoreactivity for SMI-32 and neurofilament, indicating axonal damage (Figure 2C, D). While CD8^+^ T cells were also observed between thickened axons in the lateral funiculus and adjacent to Neu-N^+^ neurons in the spinal cord (Figure 2E, F), these were rare as compared with CD4^+^ T cells.

### Cytotoxic molecules are present in the spinal cord of NF-L immunized mice

**Figure 3:**
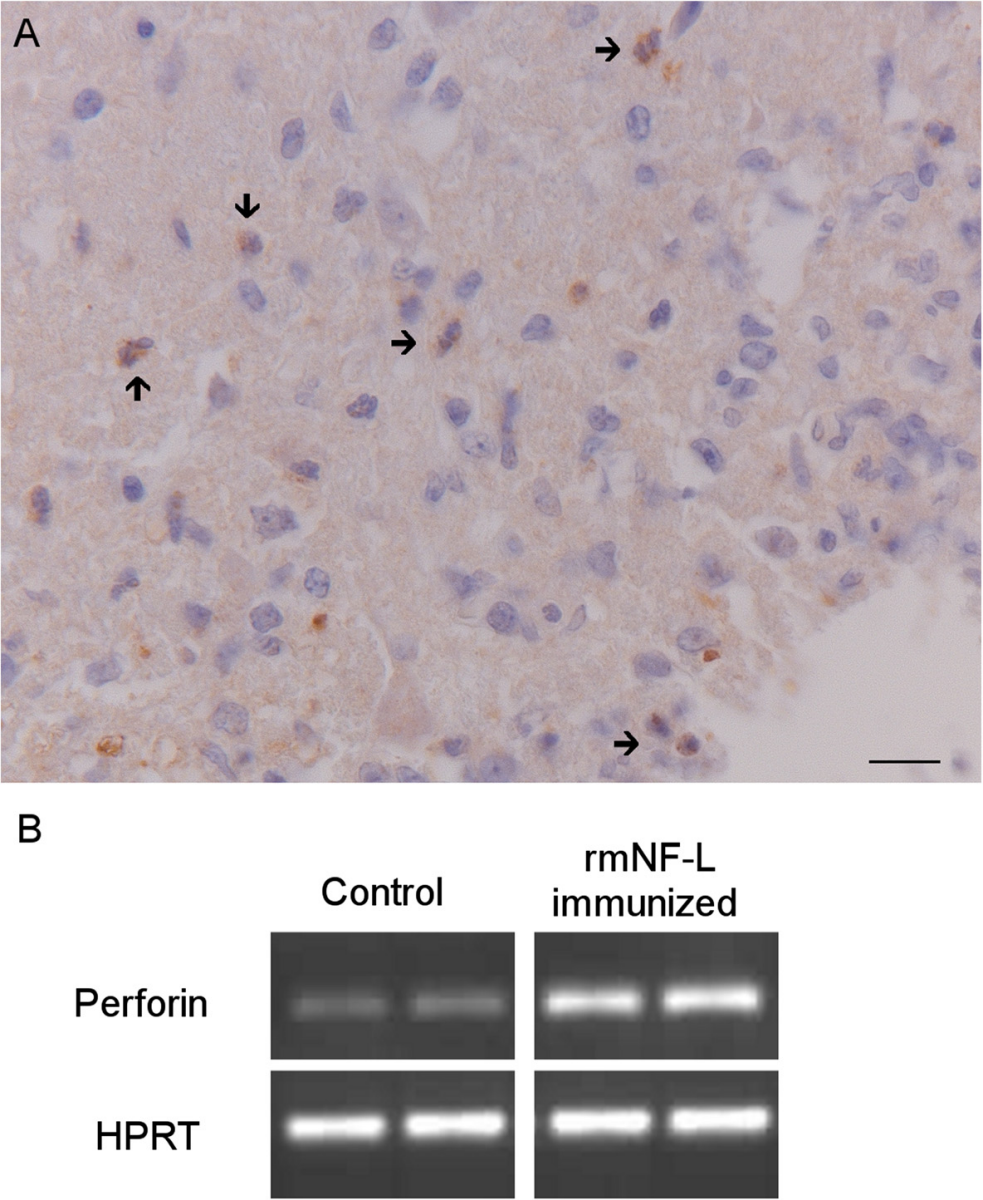
**Production of cytotoxic molecules in spastic mice. (A)** Granzyme B-positive staining (indicated by arrows) was performed on paraffin-embedded spinal cord sections: bar = 10 μm. **(B)** Gel electrophoresis of PCR products show perforin expression is increased in spastic mice (hypoxanthine guanine phosphoribosyl transferase (HPRT) = internal control). NF-L, neurofilament light.

To examine the potential mechanisms of T-cell-mediated axonal damage, the expression of the cytotoxic molecules granzyme B and perforin was examined in the spinal cord of spastic mice. Immunostaining revealed the expression of granzyme B in spinal cord lesions of mice immunized with NF-L (Figure 3A). Immunodetection of perforin in mouse CNS was not possible due to lack of specific mAbs. Instead, RT-PCR on spinal cord tissues was performed and showed that perforin mRNA levels were increased in rmNF-L immunized mice compared with controls (Figure 3B). In addition, granzyme B and perforin were expressed by peripheral CD4^+^ T cells from NF-L immunized mice compared with CD8^+^ T cells (data not shown).

### Autoimmunity to NF-L exacerbates MOG^35–55^ experimental autoimmune encephalomyelitis in ABH mice

**Figure 4:**
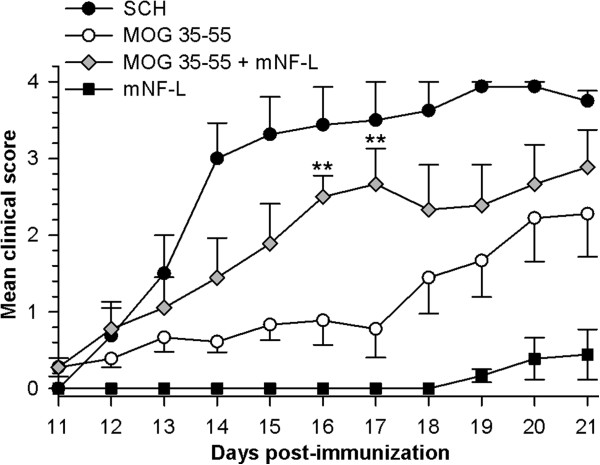
**Augmentation of MOG**^**35–55**^**-induced experimental autoimmune encephalomyelitis by neurofilament light.** ABH mice were immunized with spinal cord homogenate, MOG^35–55^, rmNF-L or rmNF-L and MOG^35–55^. **P <0.005; Mann–Whitney U test. NF-L, neurofilament light.

To increase the likelihood of exposing NF-L protein in the CNS, ABH mice were co-immunized with MOG^35–55^ to induce myelin damage (Figure 4). While SCH induced classical EAE about 11 days after immunization, co-immunization of rmNF-L with MOG^35–55^ induced significantly augmented disease in which spasticity was observed on days 16 and 17 (*P* = 0.005), indicating that myelin damage may first be necessary to expose neuronal antigens. Within this timeframe, mice immunized with rmNF-L only did not develop clinical disease as we have reported previously [7]. However, by day 21, NF-L immunized mice did develop neurological signs of disease with a mean day of onset 19.8 ± 1.6, EAE score 1.5 ± 1.0, group score 0.5 ± 0.4 and an EAE incidence of 50%.

### Immunodominant B-cell and T-cell epitopes of neurofilament light in mice

**Figure 5:**
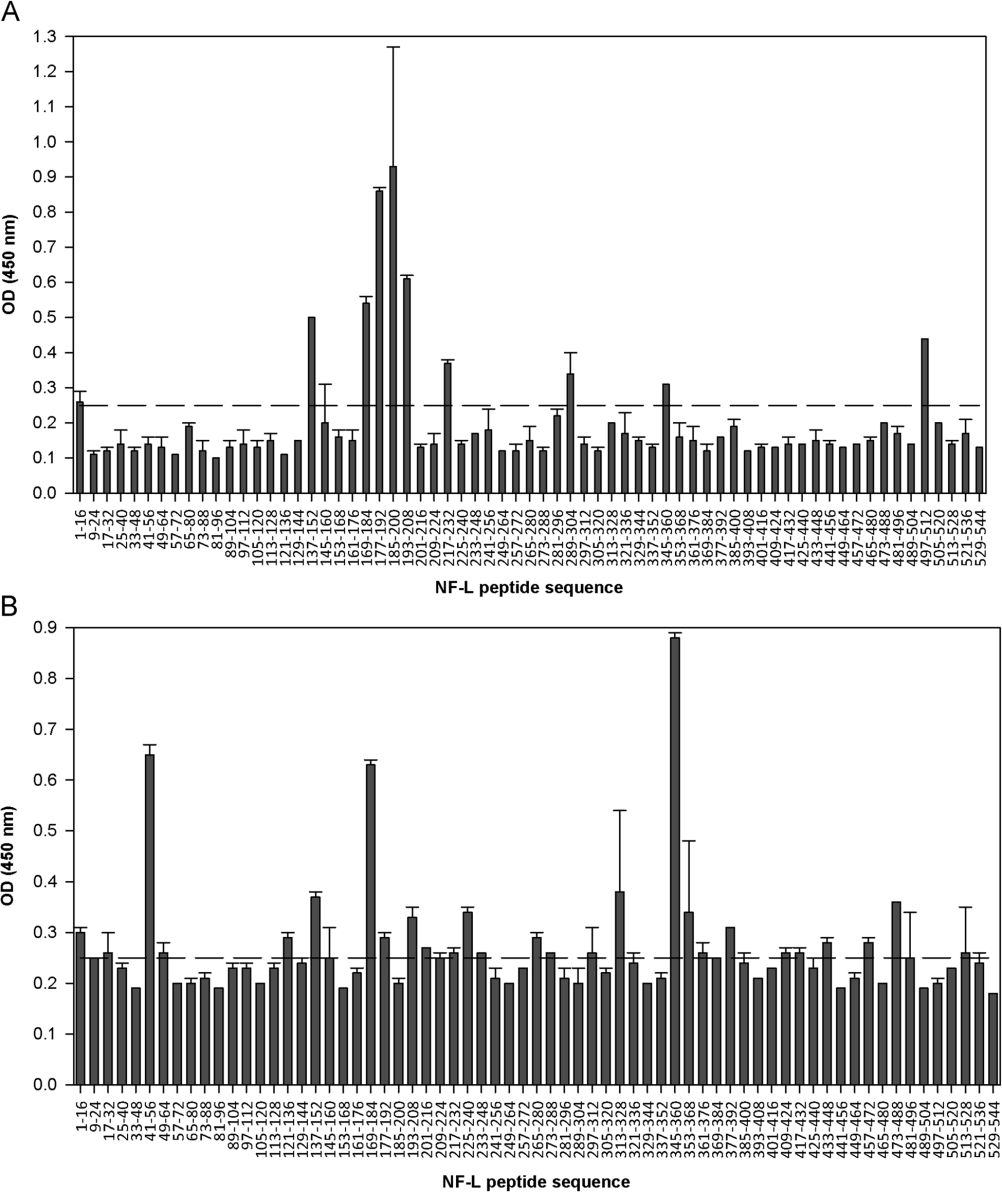
**B-cell responses to neurofilament light peptides in ABH and SJL/J mice. (A)** ABH mice and **(B)** SJL/J mice were immunized with rmNF-L and serum antibody reactivity to NF-L peptides was measured on day 10. Mean absorbance (optical density (OD) >0.25) was considered positive.

To determine immunodominant epitopes in the NF-L protein, antibody and T-cell responses to synthetic 16-mer overlapping peptides spanning the mouse NF-L sequence (Table 1) were screened in ABH and SJL/J mice immunized with rmNF-L in CFA [19]. Since we have seen before that immunization with NF-L in Biozzi mice can result in antibody production and binding to axons, it was of interest to screen the antibody responses to the overlapping peptides [7]. Antibody reactivity to NF-L peptides in both strains of mice revealed an immunodominant region that corresponds to the overlapping region (NF-L amino acids 169 to 208) and the single region (amino acids 169 to 184) in ABH and SJL/J mice respectively (Figure 5A, B). This region is located in the coil 1b domain of NF-L. In line with our observations, these motifs (amino acids 154 to 195) are likely to be linear B-cell epitopes according to the BCPred prediction algorithm [24]. In SJL/J mice, NF-L specific antibodies also reacted to the single peptides amino acids 41 to 56 and amino acids 345 to 360 (Figure 5B). In both strains, the mean absorbance values for antibody response to rmNF-L protein were within the optical density range of 1.21 to 1.34.

**Figure 6:**
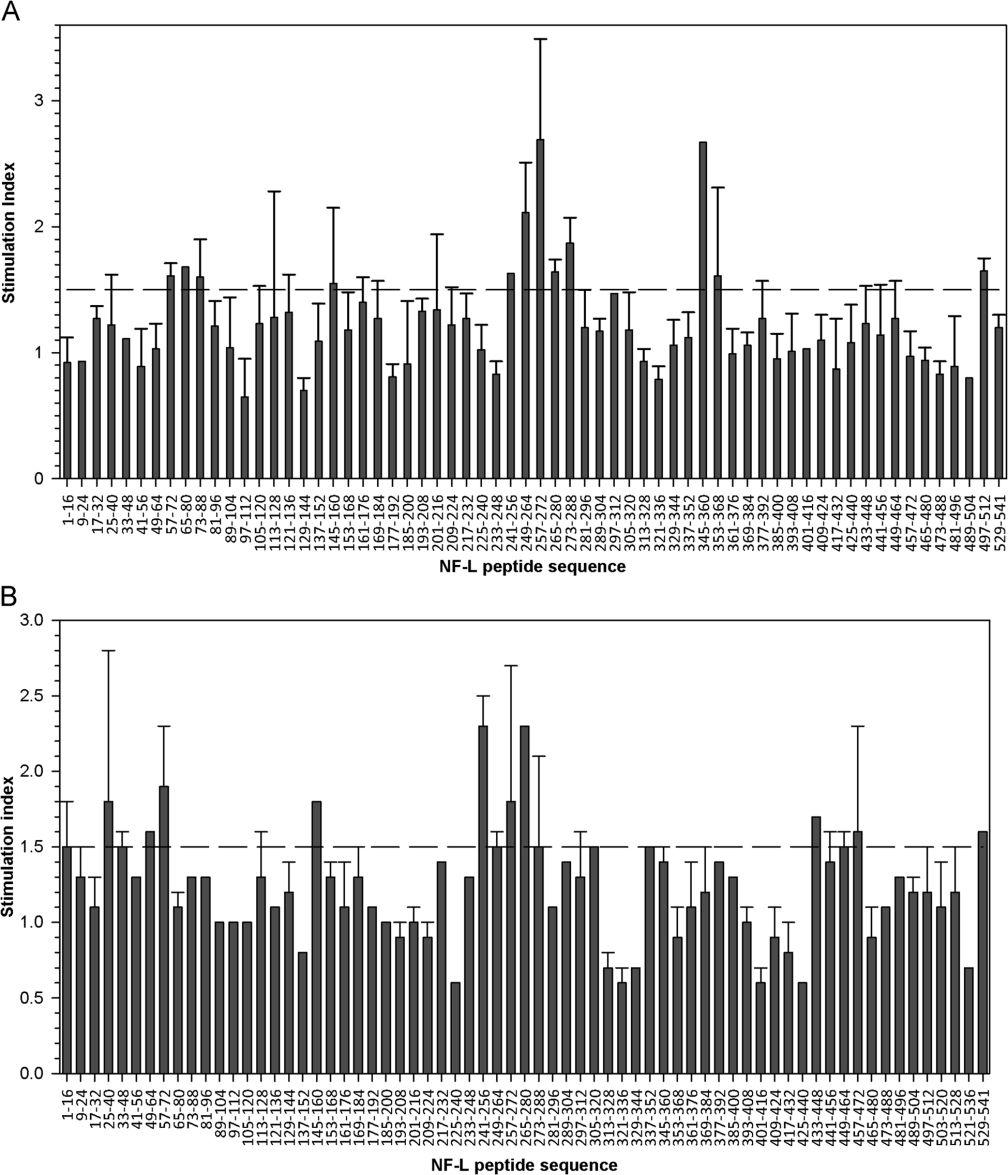
**T-cell responses to NF-L peptides in ABH and SJL/J mice. (A)** ABH mice and **(B)** SJL/J mice were immunized with rmNF-L and T-cell proliferation to NF-L peptides was measured on day 10. Stimulation index >1.5 was considered positive.

Immunodominant T-cell responses to two dominant regions, amino acids 241 to 288 and 345 to 368, were observed in ABH mice (Figure 6A). In comparison, T-cell responses in SJL/J mice were observed to amino acids 241 to 288 (Figure 6B). In both strains, the SIs were lower than responses to rmNF-L alone (SI = 5 to 10). These results are in line with data using overlapping 15-mer peptides of NF-L in ABH mice (data not shown), indicating that immunodominant epitopes reside within the rod domain (linker 2 and coil 2B) of NF-L. No overlapping peptide induced significant proliferation in cells from CFA immunized mice.

### Pathogenicity of immunodominant epitopes

To determine the pathogenicity of immunodominant epitopes, ABH mice were immunized with individual NF-L peptides spanning the T-cell immunodominant region (amino acids 241 to 272) or with a pool of immunodominant B-cell epitopes. In contrast to SCH-induced EAE, immunization of ABH mice with amino acids 241 to 256, 249 to 264, 257 to 272, 345 to 360 and 353 to 368 induced spasticity of the tail and hind limbs in line with studies using rmNF-L (Table 2, panel A) [7]. Immunization with NF-L peptides also induced a delayed onset of clinical signs occurring 10 days later than SCH-induced EAE (Table 2, panel A). Rota-rod studies did not reveal additional signs of neurological disease (data not shown). SJL/J mice immunized with rmNF-L also developed very mild clinical disease of spasticity (data not shown). The pathogenic potential of NF-L peptides was also observed with some, but not all, peptides predicted to bind with H2-A^g7^ (Table 2, panel B). No significant effect was observed after immunization with the immunodominant B-cell epitopes (amino acids 169 to 208) (Additional file 1).

**Table 2:** **Pathogenic neurofilament-light peptides in ABH mice**^**a**^

**Antigen/peptides**	**Number with EAE**	**Mean group score**^**b**^	**Mean EAE score**^**c**^	**Mean day of onset**
**Panel A**				
SCH	4/4	4.0 ± 0.0*	4.0 ± 0.0	15.3 ± 0.3
rmNF-L	4/5	0.7 ± 0.3*	0.9 ± 0.2	31.0 ± 1.4
241 to 256	1/5	0.1 ± 0.1	0.5 ± N/A	21.0 ± N/A
249 to 264	5/5	0.9 ± 0.3*	0.9 ± 0.3	25.6 ± 6.4
257 to 272	3/5	0.4 ± 0.2	0.7 ± 0.2	29.3 ± 0.6
265 to 280	0/5	N/A	N/A	N/A
273 to 288	0/5	N/A	N/A	N/A
345 to 360	3/5	0.7 ± 0.3	1.2 ± 0.3	29.3 ± 0.3
353 to 368	3/5	0.5 ± 0.2	0.8 ± 0.2	25.7 ± 4.4
**Panel B**				
129 to 144	2/5	0.2 ± 0.1	0.5 ± 0.0	18.5 ± 6.5
297 to 312	2/5	0.2 ± 0.1	0.5 ± 0.0	30.5 ± 0.5
313 to 328	0/5	N/A	N/A	N/A
113 to 128, 281 to 296, 289 to 304, 321 to 336 (4)	3/4	0.9 ± 0.4	1.2 ± 0.3	28.7 ± 0.7
201 to 216, 377 to 392, 457 to 472 (3)	0/5	N/A	N/A	N/A
**Panel C**				
CFA	0/5	N/A	N/A	N/A

^a^Animals were injected with (panel A) 200 μg spinal cord homogenate (SCH), rmNF-L or single immunodominant NF-L peptides in CFA or with (panel B) peptides in Complete Freund’s adjuvant (CFA) predicted to bind H2-A^g7^ or with (panel C) CFA alone.

^b^Mean ± standard error of the mean (SEM) of the maximum experimental autoimmune encephalomyelitis (EAE) score of animals in the group.

^c^Mean ± SEM of the maximum EAE score of affected animals.

N/A, not applicable. *P <0.05, compared with CFA immunized mice.

Additionally, the capacity of NF-L-specific T cells to transfer spasticity was tested in ABH mice. Lymph node and spleen cells from rmNF-L-immunized ABH mice were collected 12 days after immunization and stimulated for 10 days *in vitro* with rmNF-L. Activated cells were adoptively transferred into irradiated naïve recipients and followed until day 25. Mild signs of disease (score 0.5) were observed in two of seven mice revealing the pathogenic potential of T cells to NF-L (data not shown).

## Discussion

Accumulating evidence indicates that as well as myelin-specific T cells, neuronal-specific T cells may gain access to the CNS and contribute to neurodegeneration. Immune responses to neurons are reported in Rasmussen’s encephalitis [25], Alzheimer’s disease [26], Parkinson’s disease [27] and paraneoplastic disorders [5], underscoring the potential role of pathogenic neuronal-reactive T cells in MS in which axonal damage correlates with disability. To examine the role of T cells in neurodegeneration, we have studied autoimmune-meditated neurodegeneration in mice immunized with NF-L in which spasticity and paralysis, clinical features characteristic of MS, are observed [7,8]. In previous studies, infiltration of CD3^+^ and B220^+^ cells and immunoglobulin deposits were observed in spinal cord lesions of NF-L immunized mice [7].

In this study, we aimed to further investigate the cellular and humoral immune response in the mouse model of NF-L-induced neurodegeneration. Immunohistochemistry revealed the association of both CD4^+^ and CD8^+^ T cells with neuronal damage and the expression of cytotoxic molecules in the CNS. We also identify immunodominant regions and encephalitogenic epitopes on the NF-L protein. Together, our data support the accumulating evidence that T cells play a role in neuronal and axonal damage in neurodegenerative disorders. Adoptive transfer experiments to test the ability of NF-L peptide-specific T cells to induce disease would be of interest in future studies.

A pre-requisite for antigen-specific CD8^+^ cytotoxic damage of neurons is the expression of MHC class I molecules. Strong evidence exists of MHC class I on neurons, neurites and axons, revealing differential expression depending on neuronal subtype, development stage and the inflammatory stimulus. MHC class I expression is highly upregulated on neurons following IFNγ treatment but not TNFα [28]. Intriguingly, CD8^+^ T cells directed to NF-L generated from spastic mice produce high levels of IFNγ [4], indicating that T cells to NF-L could trigger progressive neurodegeneration. Further support for T-cell-mediated neuronal damage comes from the finding that, in EAE, myelin-reactive T cells are activated by neurofilament peptides [29]. Such interaction between T cells and neurons may induce antigen-specific lysis of neurons [30] or dysfunction by impairing electrical signaling [31] and induction of rapid microtubule axonal destabilization [32]. In the spastic model it is unlikely that antigen-specific CD8^+^ T-cell activation leads to neuronal damage since MHC class I was not present on neurons, either in the lesions or in normal appearing tissue. While it is unlikely that CD4^+^ T cells induce neuronal death in an antigen-specific fashion, as neurons do not express MHC class II, pathogenic CD4^+^ T cells expressing NKG2C could injury neurons and axons expressing HLA-E, as reported for oligodendrocytes in MS [33].

T-cell-mediated neuronal damage may alternatively occur via antigen-independent interactions involving Fas–FasL, TRAIL, CD11a and CD40. In culture, polyclonally activated CD4^+^ and CD8^+^ T cells are cytotoxic to human neurons [30,32], underscoring the potential pathogenic role of both CD4^+^ and CD8^+^ T cells. In line with these studies, our results show that granzyme B and perforin are present in the lesions of spastic mice, indicating that neuronal damage is more likely due to antigen-independent mechanisms. The predominance of CD4^+^ T cells in the lesions of NF-L immunized mice may be due to selective recruitment or selective depletion of CD8^+^ T cells [34]. Moreover, the ratio between the T-cell subsets in the lesions in our model is similar to myelin-induced EAE [35]. Further studies on the expression of cytotoxic molecules in T-cell subpopulations in the CNS of NF-L immunized mice will be necessary.

Our observations that co-immunization with MOG and NF-L leads to exacerbated disease is pertinent to what might occur in MS during myelin damage, in which neurons may be more vulnerable to immune responses to neuronal antigens. In this way, the course of neuronal degeneration might be accelerated in the presence of NF-L reactive T cells [4] and antibodies to NF in MS [36].

EAE studies have been instrumental in identifying the role of T cells and antibodies to myelin in disease. These have revealed important information about peptide:MHC:T-cell receptor interactions crucial for development of tolerance regimes using altered peptide ligands or tolerogenic routes for delivery of pathogenic peptides to modulate myelin-specific T cells [37,38]. These approaches have proved effective in chronic EAE models, preventing the clinical relapses, but do not control progressive disease [37], indicating that mechanisms other than myelin autoimmunity, such as autoimmunity to neuronal proteins, are involved in neurodegeneration in MS.

In ABH mice, immunization with MOG and proteolipid protein readily induces neurological disease in which flaccid paralysis and preferential myelin loss is observed. In contrast, immunization with NF-L protein induces a predominantly spastic disease in which neuronal damage is the primary pathological feature. To determine the pathogenic potential of NF-L peptide, we used a peptide mapping approach and identified a peptide core within the NF-L protein that contains similar elements to previously defined H2-A^g7^ motifs within myelin basic protein, MOG and proteolipid protein (Table 3) [19]. This supports many of the predictions made by computer modeling of peptide H2–A^g7^ interactions [39]. Similar to the finding following immunization with myelin antigens [19], several encephalitogenic epitopes were identified in NF-L immunized ABH mice. One should note that when using overlapping peptides, inappropriate sequence alignment could mask encephalitogenic epitopes or induce peptide epitopes to become tolerogenic [40]. In addition, neurofilament modulates oligodendrocyte proliferation and differentiation [41], thereby masking the possible pathogenic impact of these proteins. Peptide mapping approaches have identified immunodominant epitopes of myelin basic protein in mice and humans and prompted tolerance strategies to myelin basic protein-specific T cells in MS. Current studies are underway to identify T-cell responses to NF-L epitopes in MS patients who respond to the NF-L protein [4]. These studies may reveal whether such responses are present in subtypes of MS or correlate with extent of cognitive changes or progressive disease. Such findings may be key to the development of personal therapeutic approaches using tolerance regimens.

**Table 3:** Pathogenic peptides associated with neurological disease in ABH mice

**Protein and peptide residues**	**Amino acid sequence**	**Reference**
PLP 54 to 76	DYEYLINV**IHAF**QY**V**IGASF	[19]
MBP 12 to 35	LATASTMDHAR**HGF**LPRHRDTSGI	[19]
MOG 1 to 23	GQFRVIGPGYP**IRAL**VG**D**EQED	[19]
αB crystallin 52 to 61	FF**LRAP**SW**I**	[42]
NF-L 129 to 144	SEPSRF**RAL**YEQ**E**IRD	This study
NF-L 257 to 272	ALKD**IRA**QYEKLAAKN	This study

*MBP* myelin basic protein, *MOG* myelin oligodendrocyte glycoprotein, *NF-L* neurofilament light, *PLP* proteolipid protein.

In conclusion, we show that peptide epitopes of NF-L induce neurological disease and that potentially pathogenic CD4^+^ T cells dominate the lesions of NF-L immunized mice, a model for immune-mediated neurodegeneration. Our data suggest that, similar to peptide therapies targeting myelin responses [43], immune therapies targeting neuronal-specific T cells could thus be beneficial in reducing neurodegeneration in inflammatory disorders such as MS.

### Consent

Written informed consent was obtained from the patient for the publication of this report and any accompanying images.

## Abbreviations

BSA: Bovine serum albumin; CNS: Central nervous system; CFA: Complete Freund’s adjuvant; EAE: Experimental autoimmune encephalomyelitis; mAb: Monoclonal antibody; MHC: Major histocompatibility complex; MOG: Myelin oligodendrocyte glycoprotein; MS: Multiple sclerosis; NF-L: Neurofilament light; PBS: Phosphate-buffered saline; PCR: Polymerase chain reaction; RT: Reverse transcriptase; SCH: Spinal cord homogenate; SI: Stimulation index.

## Competing interests

The authors have no competing interests.

## Authors’ contributions

FP, BJvdS, and SA designed research, performed research, analyzed data, and wrote the manuscript. MV, MK, CB, RP-B, KU, GP, WG, RH, AR, PvdV, and DB performed research, analyzed data, and wrote the manuscript. All authors read and approved the final manuscript.

## Authors’ information

Prof. David Baker and Prof. Sandra Amor share the senior authorship.

## Supplementary Material

Additional file 1 **Figure showing the pathogenicity of immunodominant NF-L epitopes.** ABH mice (*n* = 5 per group) were immunized with NF-L peptides spanning the immunodominant regions (amino acids 241 to 288 and 169 to 208). Mice were immunized subcutaneously with 200 μg rmNF-L protein or NF-L peptides emulsified in complete Freund’s adjuvant containing *Mycobacterium tuberculosis*. Plots show the mean ± standard error of the mean daily clinical score. Click here for file
