# Supplementary figures and images for: Characterization of immune response to neurofilament light in experimental autoimmune encephalomyelitis
Source: J Neuroinflammation. 2013 Sep 22;10:118. doi: 10.1186/1742-2094-10-118 (PMC3856490; doi:10.1186/1742-2094-10-118)

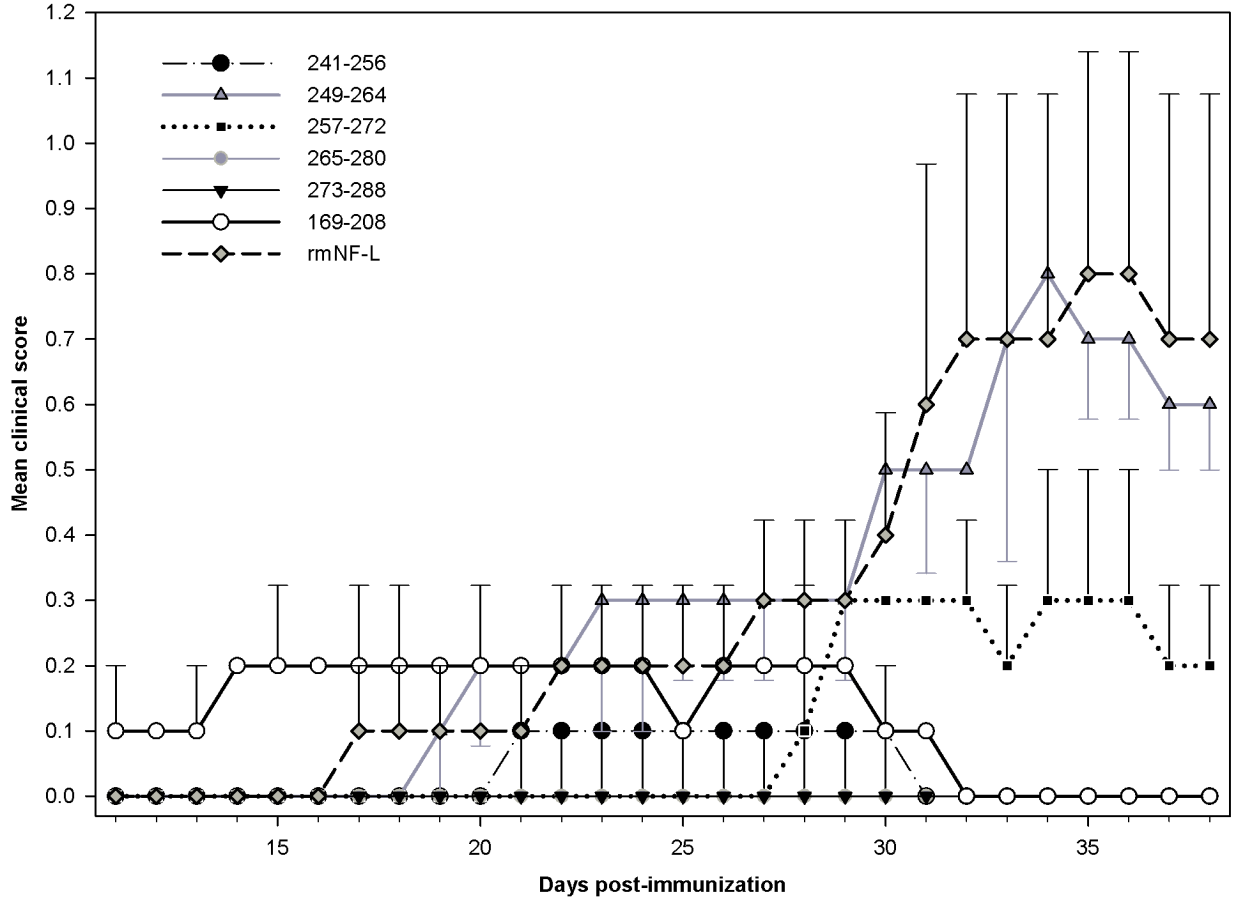

Supplement: Additional file 1 — Figure showing the pathogenicity of immunodominant NF-L epitopes. ABH mice (n = 5 per group) were immunized with NF-L peptides spanning the immunodominant regions (amino acids 241 to 288 and 169 to 208). Mice were immunized subcutaneously with 200 μg rmNF-L protein or NF-L peptides emulsified in complete Freund’s adjuvant containing Mycobacterium tuberculosis. Plots show the mean ± standard error of the mean daily clinical score. [file 1742-2094-10-118-S1.pdf]
